# Editorial: The Role of Calcium Channels in Human Health and Disease

**DOI:** 10.3389/fmolb.2022.834108

**Published:** 2022-01-21

**Authors:** Peng Zhang, Chang-Bo Zheng, Zhen Chen, Xiao-Yu Liu

**Affiliations:** ^1^ Shenzhen Key Laboratory of ENT, Institute of ENT and Longgang ENT Hospital, Shenzhen, China; ^2^ School of Pharmaceutical Science and Yunnan Key Laboratory of Pharmacology for Natural Products, Kunming Medical University, Kunming, China; ^3^ Winship Cancer Institute, Emory University, Atlanta, GA, United States; ^4^ School of Medicine, Southern University of Science and Technology, Shenzhen, China

**Keywords:** calcium channels, calcium signal, human health, human disease, therapeutic target

Calcium is one of the most common signaling molecules in cells and is involved in regulating almost all biological functions. Calcium can regulate molecular events such as gene expression and regulation, protein phosphorylation and modification; cellular activities including energy metabolism, cell division, proliferation, differentiation, and apoptosis; tissue level functions such as embryo formation and development, heart and muscle contraction, neuronal communication, learning and memory (Berridge et al., 2000; Clapham, 2007). Therefore, it is not surprising that imbalance in intracellular calcium homeostasis can lead to severe pathological disorders including cardiovascular disease and tumor (Liu et al., 2019). Calcium channels mediate the movement of calcium ions into the cytoplasm and organelles, and are important regulators of calcium homeostasis. Thus, understanding the regulatory mechanism of calcium channels is an important step towards revealing the rules that govern calcium homeostasis and life processes. This special Research Topic highlights eight studies on novel roles of calcium channels in pathophysiological processes.

Originally found in *Drosophila melanogaster*, transient receptor potential (TRP) channels are non-selective calcium channels. They are ubiquitously expressed in multiple organs and tissues of mammals, associated with cell adhesion, migration, proliferation, differentiation, apoptosis, and vascular permeability. Two original research articles in this edition of Research Topic focused on the role of TRP channels in regulating human cancers. Cai et al. demonstrated that TRPC5 protein expression on circulating tumor cells (CTC) is closely related to the tumor stage, poorly differentiated tumors, and shorter disease-free survival in radically resected colorectal cancer patients. They suggested that CTC with high TRPC5 levels could be a new indicator for clinical prognosis. Potential prognostic value was also identified for TRPM2 in a bioinformatics study of kidney renal clear cell carcinoma (KIRC) by Sun et al., where TRPM2 was shown to be involved in regulating immune cell infiltration in KIRC; providing insight for further wet-lab investigations.

Angiotensin II (Ang II) is a key bioactive molecule of the renin-angiotensin system that controls the contractility of adult cardiomyocytes, and the classical regulatory role of this molecule in the heart is fascinatingly complex (Baker et al., 1992). Qi et al. showed that extracellular Ang II can regulate calcium flow in cardiomyocytes by promoting the production of DAG and IP3. These in turn open the calcium channels TRPC3/C6, LTCC and IP3R, resulting in increased cardiomyocyte automaticity. Conversely, intracellular Ang II decreases automaticity by reducing the activity of RYR2, another regulator of calcium transport in cardiomyocytes.

Four original research articles and one review covered the role of calcium channels in regulating vascular function in this Research Topic. Liu et al. gave an outstanding review of the regulatory effects and possible mechanisms of TRPV4 in vascular dilatation, constriction, permeability, remodeling, and damage; suggesting that TRPV4 is a potential therapeutic target for vascular diseases. Xu et al. demonstrated that Orai2 enhances store-operated Ca^2+^ entry (SOCE), contributing to microvascular endothelial cell injury in an ionizing radiation (IR)-induced rat brain model. Zhang et al. explored the role of MK-626, a dipeptidyl peptidase-4 inhibitor, in hypertension and found that MK-626 restores Ca^2+^ entry mediated through Cacna1c, and increases long-coding RNAs and eNOS activity, which together improved vascular endothelial function. Guo et al. demonstrated a role for calcium signaling in IR-induced pulmonary endothelial cell ferroptosis involving the PIEZO1/Ca^2+^/calpain/VE-cadherin pathway, providing a novel target for future mitigation of IR-induced lung injury. In another IR-induced lung injury study by Huang et al., irradiation enhanced PIEZO1 and suppressed C/EBPβ expression in primary rat lung epithelial cells and the RLE-6TN lung epithelial cell line. This led to an increase in Ca^2+^ influx, activation of HIF-1α and enhancement of TGF-β1 expression, resulting in epithelial-mesenchymal transition (EMT) in these cells. The enhanced TGF-β1 expression further inhibits C/EBPβ expression, resulting in positive feedback in the PIEZO1/HIF-1α/TGF-β1/C/EBPβ axis, which the authors proposed, contribute to radiation-induced pulmonary injury and fibrosis.

In conclusion, this Research Topic highlighted studies that demonstrate the key roles played by calcium channels in human health and diseases, with possible biomarkers and therapeutic targets for consideration. However, the systematic characterization of calcium channels and their roles in calcium homeostasis is far from complete, and is a long-term goal that still requires ongoing efforts.
